# Composite Polymer Anion Exchange Membranes with Sandwich Structure and Improved Performance for Zn–Air Battery

**DOI:** 10.3390/membranes11030224

**Published:** 2021-03-22

**Authors:** Xiaoxia Cai, Yuansong Zhang, Cong Li, Guotao Zhang, Xiaotao Wang, Xian Zhang, Qiang Wang, Fuzhong Wang

**Affiliations:** 1 School of Materials Science & Engineering, Qilu University of Technology (Shandong Academy of Sciences), Jinan 250353, China; zys18765833899@yeah.net (Y.Z.); 10431200185@stu.qlu.edu.cn (G.Z.); 10431200182@stu.qlu.edu.cn (X.W.); zhangx@qlu.edu.cn (X.Z.); fullblownwang@hotmail.com (F.W.); 2 State Key Laboratory of Biobased Material and Green Papermaking, Qilu University of Technology (Shandong Academy of Sciences), Jinan 250353, China; wangqiang83@qlu.edu.cn

**Keywords:** alkaline solid polymer electrolyte, anion exchange membranes, PAA, Zn–Air battery, degrease cotton

## Abstract

In this study, we fabricated a composite polymer anion exchange membrane (AEM) with a sandwich structure. This prepared AEM demonstrated high ionic conductivity (0.25 Scm^−1^), excellent alkali resistance (8 M KOH), and good mechanical properties (tensile strength of 0.455 MPa and elongation at break of 82.13%). Here, degrease cotton (DC) treated with LiOH/urea aqueous solution was used and immersed into a coagulation bath to form a film. This film was immersed in acrylic acid (AA) monomers, and in-suit polymerization was carried out in the presence of KOH and an initiator. Finally, a composite polymer membrane with sandwich structure was achieved, in which the upper and bottom layers were mainly composed of polymerized AA (PAA) while the central layer was mainly composed of DC derived film. The central layer acted as a skeleton to improve the mechanical properties and alkali resistance. The top and bottom layers (PAA-rich layers) acted as OH- ion transport carriers, making basic cations migrate along the main chain of PAA. This newly developed composite membrane showed increased tensile strength and an elongation at break of 2.7 and 1.5 times, respectively, when compared to a control PAA/KOH AEM film. Furthermore, an electrochemical stability window of 2.0 V was measured via the cyclic voltammetry curve test, showing a wide electrochemical window and promising application in Zn–Air batteries.

## 1. Introduction

Because of their high specific capacity, high energy density, stable discharge voltage, and no pollution, Zn–Air batteries have recently received enormous attention as promising green energy devices, especially as a reliable power source for portable electronics [1,2,3,4,5]. Zn–Air batteries generally comprise Zn metal anodes, metal oxide cathodes, a potassium hydroxide (KOH)-based alkaline electrolyte solution, and separator membranes. However, there is a risk of liquid leaking and corrosivity severely restricting the practical application of such batteries. Thus, alkaline polymer electrolyte membranes with nontoxic, low leakage, and abundant raw materials with high electrical conductivity were developed [6,7,8,9,10] to replace highly corrosive liquid electrolytes. The alkaline polymer electrolyte membrane, as one of the core components of the alkaline battery, is responsible for the transfer of OH^−^ ions to form a complete battery circuit. Hence, this membrane is termed the anion exchange membrane (AEM), and is responsible for the separation of the cathode from the anode.

Over the last few decades, substantial efforts have been made to develop an anion exchange membrane with excellent electrochemical performance and distinguished reliability with regard to mechanical deformation. Since the complexes of alkali metal ions with poly(ethylene oxide) (PEO) were reported by Fenton et al. in 1973, PEO-based solid films have been considered for AEMs [11,12]. However, the crystallization induced low ionic conductivity (10^−7^ to 10^−8^ S cm^−1^ at ambient temperature) and poor mechanical properties, especially in high KOH concentration, which severely restricted the application in electrochemical devices. In order to increase ionic conductivity, a polyvinyl alcohol PVA–KOH alkaline polymer electrolyte system was developed by Lewandowski et al. [13] using ac impedance and cyclic voltammetry methods, showing a conductivity about of 10^−4^ S cm^−1^ at room temperature with poor mechanical properties. One promising approach to outperform the alkaline polymer electrolyte is to chemically cross-link the polymer matrix [14,15,16]. By introducing the cross-link bone, the electrochemical performance and mechanical properties of the alkaline polymer electrolyte can be tailored to a higher level of performance. However, cross-linked polymer membranes are prone to becoming crisp, especially in high-alkaline environments. Another effective method to improve the ionic conductivity and mechanical properties of alkaline polymer membranes is to incorporate fillers such as fiberglass [17], titanium oxide [18], chitosan [19], carbon nanotubes [20], or graphene oxide-magnetite [21] into the PVA polymer matrix.

Cellulose has recently been considered an effective filler in electrochemical polymer matrices. Qing et al. [22] added 3 wt% cellulose nanofibrils into a poly (acrylic acid) (PAA) matrix and observed enhanced ionic conductivity and mechanical performance when compared with pristine PAA. Zhang et al. [21] prepared a laminated, cross-linked nanocellulose/graphene oxide (GO) membrane functionalized with quaternary ammonium (QA) for a flexible rechargeable Zn–Air battery. The QA-functionalized nanocellulose/GO (QAFCGO) membrane showed superior OH-conductivity at room temperature.

In our previous work, we also reported that modified microcrystalline cellulose composited with PVA/PEO can increase alkali resistance, ionic conductivity, and mechanical properties simultaneously [4,23]. After the addition of 70 wt% KOH and 5 wt% LD-MCC (LiCl/DMAc treated MCC), this PVA/PEO/MCC composite electrolyte demonstrated an improved ionic conductivity of 0.153 Scm^−1^ compared to the pristine sample [4]. In conclusion, cellulose is favorable for the improvement of the ionic conductivity and alkali resistance of polymer electrolytes. However, our previous study showed that the addition of more MCC would result in the creation of an MCC aggregate in the electrolyte membrane which impaired the battery performance and caused a decrease in the ionic conductivity.

Considering the issue mentioned above, a polymer electrolyte membrane with a sandwich structure was developed in this paper. Instead of the commonly used MCC, regenerated degreasing cotton (RDC) was selected. Successful polymerization of the acrylic acid on the surface of the RDC network (soaked by KOH solution) was achieved. The resultant poly-acrylic potassium (PAAK) served as the outer layers, and the RDC network acted as the inner layer, yielding an alkali polymer electrolyte membrane with a sandwich structure. This design avoids the issue of MCC aggregation by directly using the RDC network as the inner layer to support the entire membrane in the alkali environment. On the other hand, the PAAK outer layers can be used as an effective conducting phase for ion transmission. The structure and performance of this polymer electrolyte membrane were studied in this paper to show the possibility of its use in Zn–Air batteries.

## 2. Materials and Methods

### 2.1. Materials

Degreasing cotton (DC, Chemical Reagent Factory of Tianjing, Tianjing, China), urea (AR, the Reagent Factory of Tianjing, Tianjing, China), LiOH (AR, Sigma Aldrich Corp., St. Louis, MO, USA), *N*,*N*′ methylene-bisacrylamide (MBA, Chemical Reagent Factory of Tianjing, Tianjing, China), acrylic acid (AA, Chemical Reagent Factory of Tianjin, Tianjing, China), KOH (AR, the Reagent Factory of Tianjing, Tianjing, China), K_2_S_2_O_8_ (AR, Sigma Aldrich Corp., Shanghai, China), and ethyl alcohol (AR, Chemical Reagent Factory of Tianjing, Tianjing, China) were used as received.

### 2.2. Preparation of RDC/PAAK/KOH Alkaline Polymer Electrolyte Membranes

**Figure 1:**
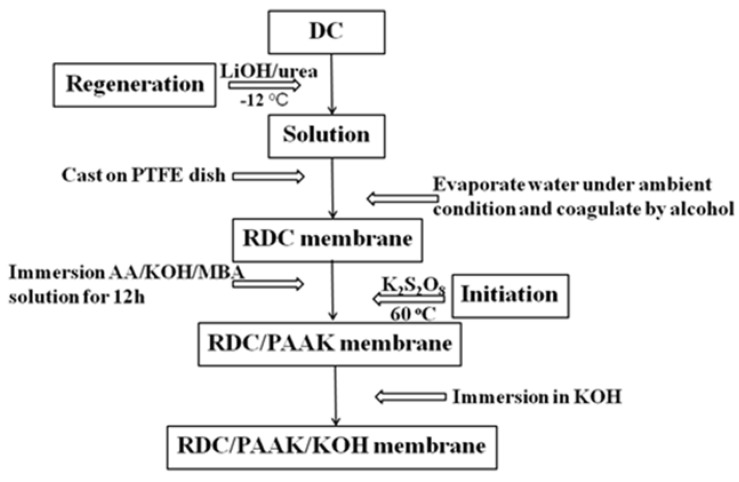
Schematic diagram for the preparation of alkaline regenerated degreasing cotton/poly-acrylic potassium/potassium hydroxide (RDC/PAAK/KOH) composite membranes.

Figure 1 schematically illustrates the preparation process of the RDC/PAAK/KOH composite films. Three steps were implemented in the preparation of the membranes: (i) The DC was treated with urea/LiOH solution and freezing-thawing to obtain an aqueous solution. The aqueous solution was then poured into a PTFE dish and evaporated at ambient temperature. Finally, a membrane, named RDC, was coagulated by ethyl alcohol for later use; (ii) The RDC membrane was immersed into an aqueous solution consisting of 10 mL AA, 8 g KOH, 0.15 g MBA, and 10 mL ultrapure water for 12 h. Then, 1.5 wt% K_2_S_2_O_8_ solution was sprinkled on the surface of the membrane as an initiator. Finally, the membrane was placed in an oven for polymerization at 60 °C for 2 h to acquire an RDC/PAAK membrane; and (iii) The RDC/PAAK membrane was soaked in KOH solution with a concentration of 8 M for 3 h, and then kept in a humidity controlled oven to maintain equilibrium. The resultant membrane is named RDC/PAAK/KOH (8 M)-3 h. Similarly, the RDC/PAAK membranes soaked in various concentrations of KOH at different times are named RDC/PAAK/KOH (x M)-x h.

### 2.3. Zn–Air Battery Design and Assembly

Preparation of air electrode: the carbon slurry for the active layer of the air electrode was prepared based on a mixture of 10 wt% acetylene black, 40 wt% KMnO_4_, 10 wt% Vulcan XC-72R, 40 wt% Na_2_SO_4_, and an appropriate amount of PVDF dissolved in ethyl alcohol. The mixture was then coated on nickel foam under a pressure of 80 kg/cm. The thickness of the air electrode was maintained within a range of 0.6 to 0.8 mm.

Preparation of Zn electrode: the Zn electrode was prepared by mixing Zn powder and a certain amount of PVDF with the aid of N-methyl-pyrrolidone to obtain a homogeneous, sticky substance. Then, the sticky product was coated on the other nickel foam under a pressure of 80 kg/cm^2^. Both Zn and air electrodes were placed in an oven at 100 °C in order to remove the residual solvent.

**Figure 2:**
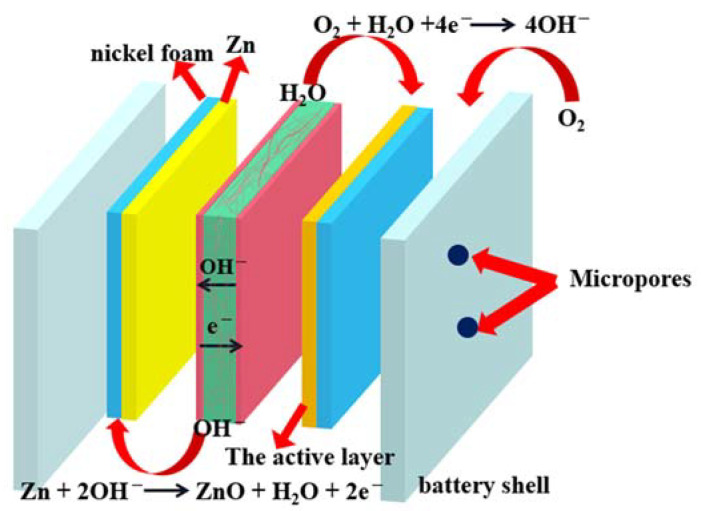
Assembly of Zn–Air battery with RDC/PAAK/KOH alkaline polymer electrolyte membrane.

The assembly of the alkaline polymer electrolyte Zn–Air battery is schematically represented in Figure 2, in which the solid-state Zn–Air cell consisted of a Zn electrode, RDC/PAAK/KOH alkaline polymer electrolyte, and air electrode. The above three parts were arranged and packaged into battery shells with micropores for the circulation of oxygen.

### 2.4. General Characterization

The morphology of the RDC/PAAK/KOH alkaline polymer electrolyte membrane was investigated by scanning electron microscopy (SEM, JSM-7500F, JEOL Ltd., Tokyo, Japan). The freeze-dried specimens were fractured by liquid nitrogen and then coated with gold for observation.

Wide-angle X-ray diffraction (WAXD) measurements were carried out on an XRD diffractometer (XRD-6100, SHIMADZU Ltd., Tokyo, Japan). The patterns with Cu Kα radiation at 40 kV and 30 mA were recorded in the 2θ region from 10° to 70°. Samples were soaked in liquid nitrogen for 10 min and then ground into pieces for the WAXD test.

The molecular structures of alkaline polymer electrolyte membranes were characterized by Fourier-transform infrared (FTIR) spectroscopy (NICOLET iS10, Madison, WI, USA) with ATR mode. Freeze-dried samples were ground into pieces and the IR spectra were recorded with a wavenumber resolution of 4 cm^−1^ in the range of 500–4000 cm^−1^. Here, air was selected as the background reference.

Thermogravimetry analysis (TGA) and derivative thermogravimetry (DTG) of the polymer electrolyte membranes were performed using a TGA1 STARe system apparatus (TG7, METTLER TOLEDO, Zurich, Switzerland). Samples of about 10 mg were loaded into an aluminum pan and then heated from 50 to 700 °C at a rate of 10 °C min^−1^ under a nitrogen atmosphere.

The ionic conductivity of the RDC/PAAK/KOH alkaline polymer electrolyte was measured by an AC impedance method using an electrochemical impedance analyzer (CHI660E, Shanghai Chenhua Instruments Co., Shanghai, China) at room temperature, and the AC frequency was scanned from 10^5^ to 10^−2^ Hz with an amplitude of 5 mV. The samples with a diameter of 1 cm were sandwiched between two plates of stainless steel with a surface area of 0.785 cm^2^ (SS|SPE|SS). The bulk resistance (Rb) was determined from the cross point of the Nyquist curve at the real axis. The calculation formula of ionic conductivity is σ = L/(Rb × A), where L, Rb, and A represent thickness (cm), bulk resistance (ohm), and area (cm^2^) of the sample, respectively.

The electrochemical stability window was determined by a cyclic voltammetry curve which was tested using a CHI660E Electrochemical Workstation. The samples with a radius of 0.5 cm were placed between two plates of stainless steel (SS|SPE|SS). RDC/PAAK/KOH alkaline polymer electrolyte membranes were cycled in the voltage region of −1.5 V to 1.5 V with a scan rate of 10 mV s^−1^ at 25 °C.

The charge–discharge performance of the battery was tested using LAND autocycler (CT2001A, Wuhan Blue Electrical Co., Wuhan, China). The extent of electric current for charging and discharging of Zn–Air cells was determined according to a calculation method J = (m − m0)*NC/A. Here, J was current density, m and m0 were the weight (g) of Zn electrode and nickel foam, respectively, N was multifarious values, C was theoretical specific capacity, and A was the area of alkaline polymer electrolyte membrane for Zn–Air battery.

The mechanical properties of RDC/PAAK/KOH alkaline polymer electrolyte membranes were evaluated by a mechanical testing machine (WDL-005, Jinan Xinshijin Experimental Instrument Co., Jinan, China) with a crosshead speed of 20 mm/min. Specimens with a typical size of 30 mm × 11 mm (length × width) were used and coated with silicone wax to avoid water evaporation during the experiment.

## 3. Results

### 3.1. Morphology and Structure Analyses

**Figure 3:**
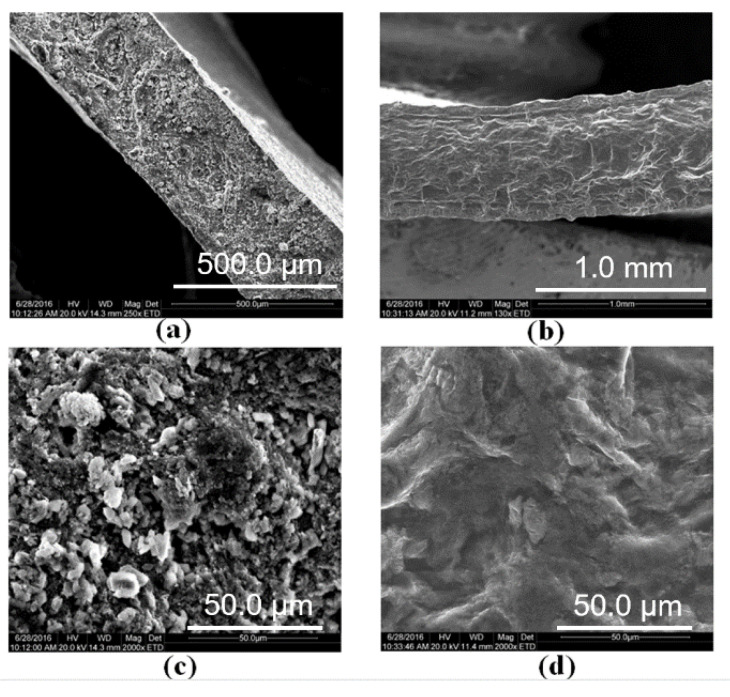
SEM microphotographs of the cross-sections for alkaline polymer electrolyte membranes of (**a**) RDC/KOH (8 M)-3 h and (**b**) RDC/PAAK/KOH (8 M)-3 h; (**c**) and (**d**) are the enlarged areas of the cross-sections for RDC/KOH (8 M)-3 h and RDC/PAAK/KOH (8 M)-3 h, respectively.

SEM measurements were conducted to reveal the morphologies of RDC/KOH polymer electrolyte membranes. The cross-sections of the RDC/KOH (8 M)-3 h and RDC/PAAK/KOH (8 M)-3 h membranes are displayed in Figure 3a,b, respectively. Compared to the RDC/KOH (8 M)-3 h membrane, a sandwich structure with two outer layers of about 0.1 mm thickness can be discerned on the cross-section of the RDC/PAAK/KOH (8 M)-3 h membrane. The formation of this sandwich structure can be attributed to the polymerization of acrylic potassium on the surface of the RDC, by which a surface layer, consisting of the polymerized acrylic potassium (PAAK), was achieved. Moreover, it is noteworthy that compared to the RDC/KOH (8 M)-3 h membrane, after the introduction of AA, no remarkable KOH aggregates were found in the inner layer of the RDC/PAAK/KOH (8 M)-3 h membrane (Figure 3c,d). This phenomenon can be attributed to the PAA components formed via the polymerization of AA monomers in the RDC membrane matrix by which the carboxylic groups on the PAA chains would facilitate the interaction with KOH and improve the homogenous dispersion of KOH in the polymer matrix. It can be speculated that the improved dispersion of KOH in the inner layer of the electrolyte membrane would benefit the ionic conductivity. Moreover, PAAK outer layers would play an important role as the continuous phase for the ionic conduction. A remarkable performance improvement can be imagined for the electrolyte membrane with sandwich structure compared to the one-layer counterpart.

**Figure 4:**
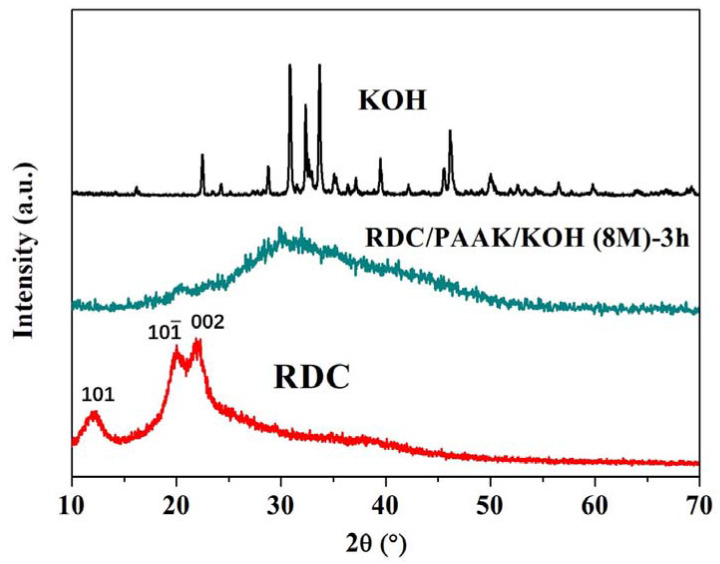
WAXD profiles for RDC, RDC/PAAK/KOH (8 M)-3 h, and neat KOH at 2θ ranging from 10° to 70°.

In order to explore the crystallinity of the alkaline polymer electrolyte membranes, WAXD was used in the experiment (Figure 4). For the RDC sample, three distinct peaks at 2θ = 12, 20°and 22° were recognized, indicating the typical absorption signals of cellulose II crystal planes (101), (10$\bar{1}$), and (002). After the introduction of PAAK, these two typical peaks (i.e., 2θ = 20° and 2θ = 22°) vanished as the WAXD profile of RDC/PAAK/KOH (8 M)-3 h showed. This result indicated that PAAK reduced the crystallinity of the RDC. Moreover, a broad peak was found at around 2θ = 30° for the RDC/PAAK/KOH (8 M)-3 h. This peak can be attributed to the KOH component as evidenced by the typical crystal signals at 2θ ranging from 30°to 35° for the neat KOH. Compared to the sharp peaks of the neat KOH, the broad peak indicates the low crystallinity degree and imperfect crystal of KOH in the RDC/PAAK/KOH (8 M)-3 h membrane. This result is consistent with the morphologies observed in Figure 3 in which no obvious KOH aggregate was observed due to the homogenous dispersion of KOH in the membrane matrix.

**Figure 5:**
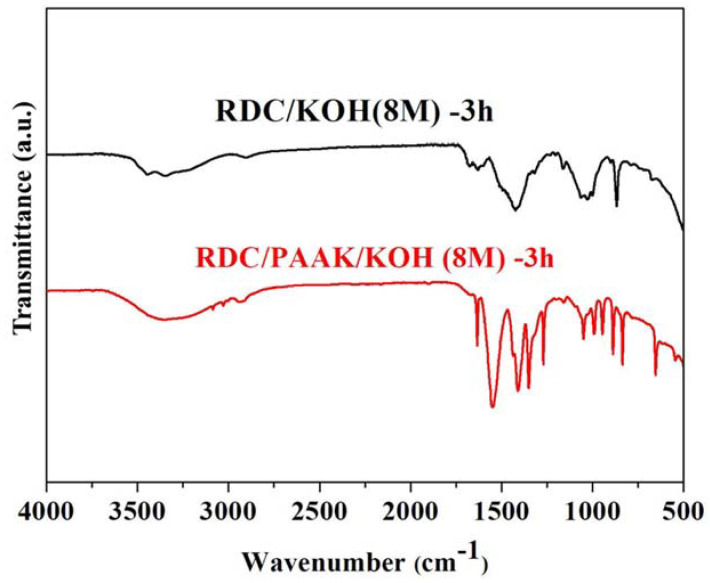
FTIR spectra for RDC/KOH (8 M)-3 h and RDC/PAAK/KOH (8 M)-3 h.

Structural features of the membranes were further revealed via Fourier-transform infrared (FTIR) spectroscopy. Distinct IR features between samples are displayed in Figure 5. Compared to RDC/KOH (8 M)-3 h, a sharp absorption peak at 1700 cm^−1^ was observed for RDC/PAAK/KOH (8 M)-3 h. This signal was attributed to the carboxyl groups of the acrylic acid, indicating the successful introduction of AA into the polymer matrix. This carboxyl group is beneficial for OH^−1^ absorption, and was further confirmed by the broad absorption band at around 3300 cm. This band was attributed to the –OH vibration which is a combination of the –OH groups of KOH and cellulose. Compared to RDC/KOH (8 M)-3 h, it is noteworthy that the band was much stronger for RDC/PAAK/KOH (8 M)-3 h, indicating that more KOH had been absorbed by the polymer matrix. Combined with the homogenous dispersion of KOH in the membrane matrix as observed in Figure 3d, it can be speculated that the introduction of PAA within the polymer matrix and its interaction with the KOH enhance the KOH retention capacity and facilitate the ionic conductivity for the electrolyte membrane.

### 3.2. Property Evaluation

#### 3.2.1. Mechanical and Thermal Properties

**Figure 6:**
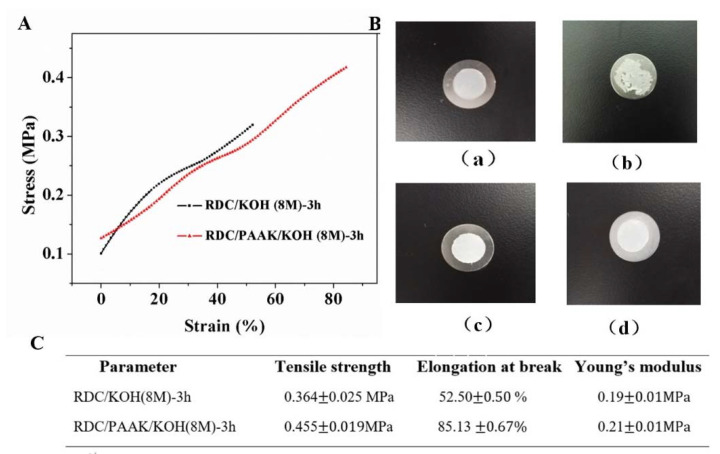
(**A**) The stress-strain curves of the prepared electrolyte membranes; (**B**) Pictures showing the states of the membranes for RDC/KOH (8 M)-3 h before (**a**) and after (**b**) being clamped and RDC/PAAK/KOH (8 M)-3 h before (**c**) and after (**d**) being clamped; (**C**) Specific mechanical values for RDC/KOH (8 M)-3 h and RDC/PAAK/KOH (8 M)-3 h.

Mechanical properties also played an important role in the application of alkaline polymer electrolyte membranes. Figure 6A demonstrates the mechanical properties of the prepared alkaline polymer electrolyte membranes. After the introduction of PAAK for RDC/KOH (8 M)-3 h alkaline electrolyte membrane, both the tensile strength and elongation at break improved remarkably. Specific values of the tensile strength, elongation at break, and Young’s modulus are displayed in Figure 6C. When the alkaline polymer membranes were pressed by battery clamps (Figure 6B(b)), it was found that RDC/KOH (8 M)-3 h membrane was crushed to pieces (Figure 6B(b)) while the RDC/PAAK/KOH (8 M)-3 h membrane kept intact (Figure 6B(d)). Clearly, the introduction of the PAAK significantly enhanced the mechanical properties of the RDC-based electrolyte membrane. As the homogenous morphology observed in the inner layer of the RDC/PAAK/KOH (8 M)-3 h membrane (Figure 3d), PAAK dispersed and penetrated into the RDC matrix and combined with the RDC network to form a coherent whole composite. This composite possessed higher mechanical strength in comparison with the RDC/KOH network without PAAK. On the other hand, the RDC network was also beneficial for the PAAK in terms of alkali-resistance. As Figure 6A shown, after undergoing a soak by 8 M KOH solution for 3 h, the sandwich membrane with PAAK as the outer layers still demonstrated a good mechanical strength (0.455 MPa) and high elongation at break (85%).

**Figure 7:**
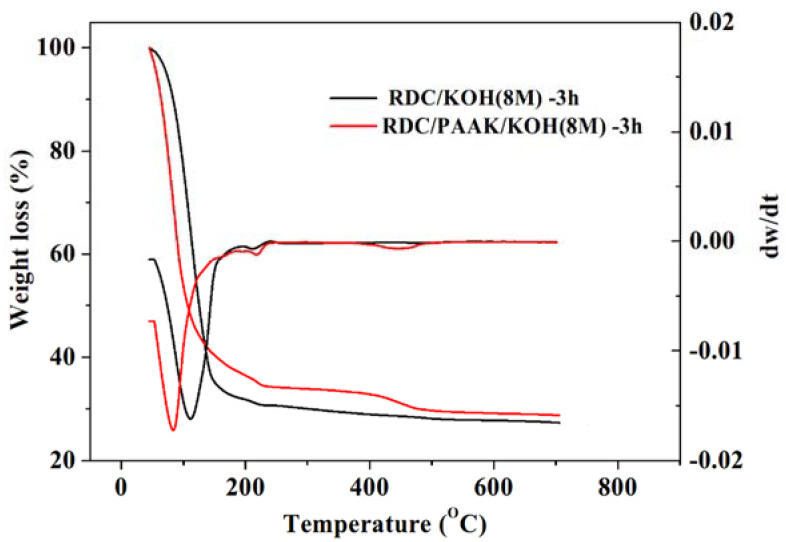
The TGA curves for RDC/KOH (8 M)-3 h and RDC/PAAK/KOH (8 M)-3 h alkali polymer electrolytes, respectively.

TGA curves demonstrated distinct weight-loss behaviors between the RDC/KOH (8 M)-3 h and the RDC/PAAK/KOH (8 M)-3 h samples (Figure 7). Combined with the DTGA curves, three weight-loss stages can be recognized on the curve of the RDC/PAAK/KOH (8 M)-3 h, while two weight-loss stages were recognized for the RDC/KOH (8 M)-3 h. The first stage ranging from 50 to 200 °C for both samples is attributed to the water loss. It can be found that compared to the RDC/KOH (8 M)-3 h, RDC/PAAK/KOH (8 M)-3 h showed a faster water loss behavior, indicating the water adsorption strength in the RDC/PAAK/KOH (8 M)-3 h membrane was weaker than that in the RDC/KOH (8 M)-3 h membrane. Two similar weight-loss peaks at around 250 °C on the DTGA curves were observed for these two samples, and the peaks were attributed to the thermal degradation of RDC. A distinct weight-loss peak at 450 °C can be recognized on the DTG curve for RDC/PAAK/KOH (8 M)-3 h. This peak can be attributed to the thermal degradation of PAA. Above 500 °C, no obvious weight-loss was observed, and the remained component was attributed to the residual KOH. It can be found that the remained KOH content is 32% for RDC/PAAK/KOH (8 M)-3 h which is higher than the remained KOH content of 28% in the RDC/KOH (8 M)-3 h sample. The higher KOH content in the RDC/PAAK/KOH (8 M)-3 h system indicated higher KOH retention capacity and possible better ionic conductivity for the alkali polymer electrolyte.

#### 3.2.2. Electrochemical Properties

**Figure 8:**
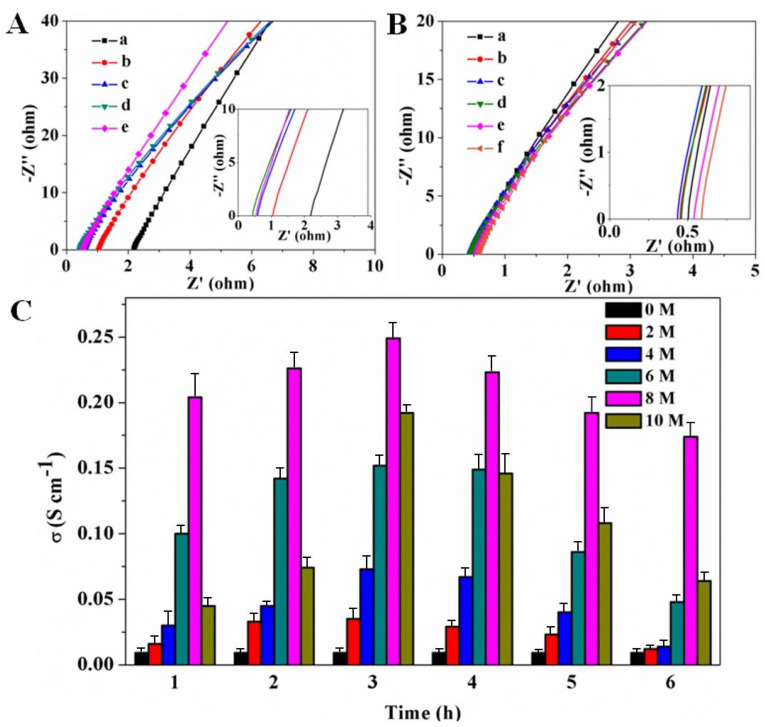
The AC impedance spectra of (**A**) RDC/PAAK/KOH -3 h with various concentration KOH solution of (**a**) 2 M, (**b**) 4 M, (**c**) 6 M, (**d**) 8 M, and (**e**) 10 M; (**B**) RDC/PAAK/KOH (8 M) with various soaking times of (**a**) 1 h, (**b**) 2 h, (**c**) 3 h, (**d**) 4 h, (**e**) 5 h, and (**f**) 6 h for alkaline polymer electrolytes; (**C**) ionic conductivities of RDC/PAAK/KOH alkaline polymer electrolytes with various concentrations of KOH and soaking time.

All the alkaline polymer electrolyte membranes were measured by AC impedance at ambient temperature. Based on the AC impedance spectra (Figure 8A,B), the bulk resistance Rb of the alkaline polymer electrolyte membranes can be obtained. Figure 8A showed that the Rb was in the order of 0.4–2.2 Ω for alkaline polymer electrolyte membranes which were soaked by KOH solution with various alkaline concentrations. In contrast, Figure 8B showed the Rb was in the range of 0.4–0.7 Ω upon undergoing different immersing time. The relationship between ionic conductivity σ and the impedance Rb is characterized via Equation (1).

σ = L/(Rb × A)(1)

Figure 8C exhibited the ionic conductivities for all the alkaline polymer electrolyte membranes. It can be found the ionic conductivity increased first and then decreased with the increase of the KOH solution concentration. The same tendency was observed for the ionic conductivity when the soaking time increased. The highest ionic conductivity was 0.25 S cm^−1^ which was obtained when an 8 M KOH solution and 3 h soaking time were applied in the preparation of the electrolyte membrane.

**Figure 9:**
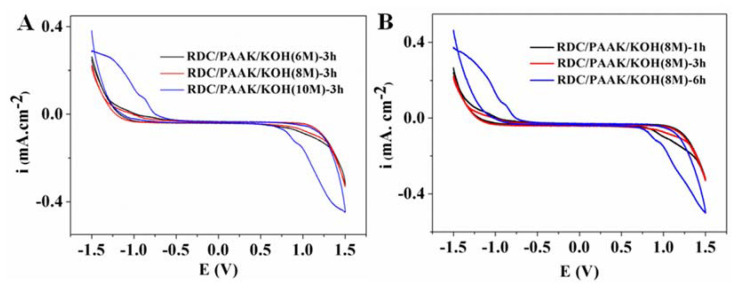
The cyclic voltammetry curves of (**A**) RDC/PAAK/KOH -3 h alkaline polymer electrolyte membranes with various concentration KOH solution and (**B**) RDC/PAAK/KOH (8 M) alkaline polymer electrolyte membranes with different soaking times.

In addition to high ionic conductivity, the wide electrochemical stability window was also an important factor for the alkaline polymer electrolyte membrane in the application of Zn–Air batteries. The electrochemical stability window was defined as a potential platform area where no induced current occurs with the variation of the voltage. The cyclic voltammetry curves for the RDC/PAAK/KOH alkaline polymer electrolytes with different KOH concentrations and soaking times are shown in Figure 9. It can be found an electrochemical stability window of about 2.3 V was obtained for the RDC/PAAK/KOH electrolytes. Though the width of the stability window barely reduced with the increase of the KOH concentration and the soaking time, a value of about 2 V was still available for the alkaline electrolytes. Clearly, the good alkali resistance was attributed to the RDC network laminated in the inner layer of the sandwiched electrolyte. This RDC network acted as a role of the skeleton structure, avoiding the collapse of the PAAK outer layers and supporting the entire sandwiched membrane even in a high-concentration alkali environment. Simultaneously, the PAAK outer layers, together with the PAAK phase, dispersed in the inner layer were responsible for the ion transmission. These results indicate that the sandwiched RDC/PAAK/KOH electrolytes possess a relatively stable voltage and a high ionic conductivity simultaneously, showing promising potential in the application of Zn–Air batteries.

#### 3.2.3. Performance of Zn/ASPE/Air Battery

**Figure 10:**
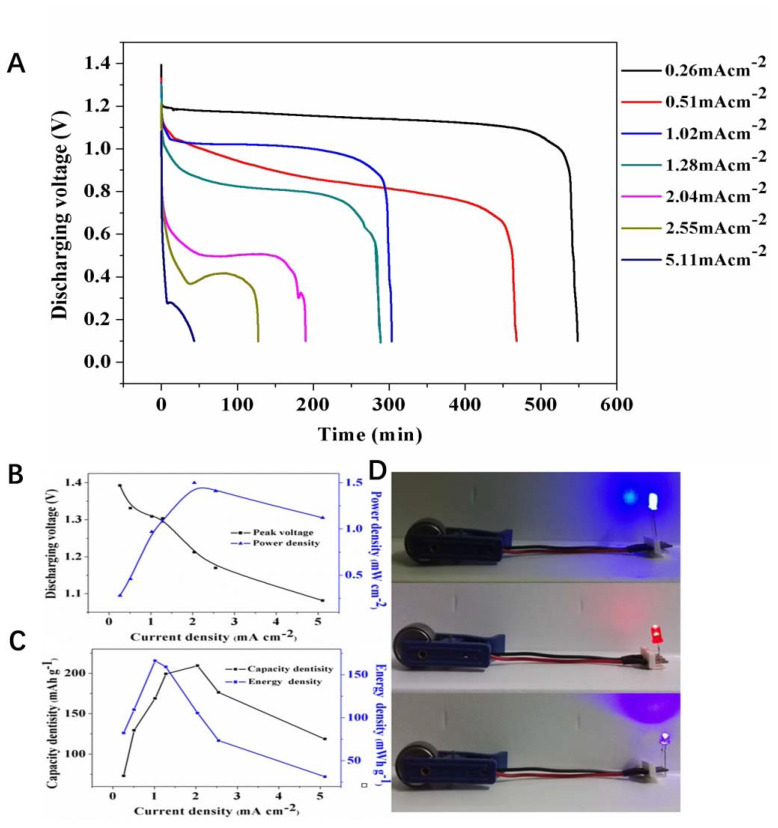
(**A**) Discharge curves versus constant current densities for Zn–Air batteries with RDC/PAAK/KOH (8 M)-3 h alkaline polymer electrolyte membranes. (**B**) The discharging voltage and power density profiles versus current density. (**C**) Capacity and energy density profiles versus current density (**D**) Luminescence experiment of the LED bubbles via Zn–Air batteries with RDC/PAAK/KOH (8 M)-3 h alkaline polymer electrolyte membranes.

The performance of RDC/PAAK/KOH (8 M)-3 h electrolyte membrane in the application of Zn–air batteries was conducted via discharge experiment. Here, the discharge curves of Zn–air batteries with different current densities ranging from 0.26 mAcm^−2^ to 5.11 mAcm^−2^ were measured by LAND autocycler (Figure 10A). A voltage platform lasting 500 min can be recognized when the current density is 0.26 mAcm^−2^. With the increase of the current density, the width of the voltage platform kept decreasing. No voltage platform can be found in Figure 10A when the current density is higher than 5 mAcm^−2^. This phenomenon implies the rapid consumption of Zn electrodes with the increase of the current density. In addition, it can be speculated from the discharge curves that a relatively good diffusion of OH^−1^ in the alkaline electrolyte membrane can be achieved when the current density is no higher than 0.26 mAcm^−2^. As for a current density higher than 5 mAcm^−2^, the RDC/PAAK/KOH-based alkali electrolyte would cause an insufficient OH^−1^ transmission and lead to a rapid voltage decline. The variation of the power density, capacity density, and energy density with the current density are displayed in Figure 10B,C. It can be seen that the power density and energy density obtained the maximum values when the current density reached 2.04 mAcm^−2^, while the maximum capacity density appeared when the current density reached 1.02 mAcm^−2^. The luminescence experiment was conducted as Figure 10D shown, in which a LED bubble was connected with the prepared Zn–Air battery and the glowing time lasted about 15 h. Based on the RDC/PAAK/KOH system, the alkali polymer electrolyte membranes demonstrate a promising potential in the application of Zn–Air battery. On the other hand, higher current density endurance and powder density are necessary to improve the battery performance, and further efforts are required in future.

## 4. Conclusions

A novel sandwiched alkaline polymer electrolyte membrane with high ionic conductivity and alkali resistance was prepared by solution-casting and solution polymerization approaches. The inner layer RDC served as the framework to improve the mechanical properties and alkali resistance, while the outer layers PAAK served as the conducting phase for ion transmission. The results showed that after the introduction of PAAK into the polymer electrolyte, the elongation at break increased from 52.50% to 82.13%, and the tensile strength was enhanced from 0.364 MPa to 0.455 MPa. A high ionic conductivity (0.25 Scm^−1^) and alkali resistance (8 M KOH) were achieved for the polymer electrolyte membrane. Furthermore, an electrochemical stability window of 2.0 V was measured via the cyclic voltammetry curve test. The prepared electrolyte membranes with the RDC/PAAK/KOH-based sandwich structure show promising potential in the application of Zn–Air batteries.

## Data Availability

Not applicable.
